# The mediating role of activity attachment in the relationship between gardening frequency, leisure orientation, and mental wellbeing: evidence from resident gardeners with implications for future gardening tourism

**DOI:** 10.3389/fpsyg.2026.1767661

**Published:** 2026-02-20

**Authors:** Gulnara Mamirkulova, Rashid Menhas

**Affiliations:** 1 School of Business, Shandong Xiehe University, Jinan, Shandong, China; 2 School of Nursing, Shandong Xiehe University, Jinan, China

**Keywords:** activity attachment, ecotherapy, Kazakhstan, leisure orientation, mental wellbeing, public health, urban gardening

## Abstract

**Background:**

As modern urbanization, digitalization, and technological progress increasingly separate people from their natural environment, engaging with nature to improve residents’ wellbeing is becoming an increasingly challenging task.

**Purpose:**

By applying the most accessible therapeutic effect of gardening, our study aims to explore how the frequency of gardening activities and leisure orientation contribute to attachment to the activity. In turn, attachment to an activity increases mental wellbeing and reduces stress levels. Our case study was conducted in the developing urban area of Shymkent in Kazakhstan.

**Methods:**

Using snowball sampling, we surveyed 210 urban residents, of whom 135 were gardeners and 75 were non-gardeners. The survey measured gardening frequency, leisure orientation, activity attachment, mental wellbeing and perceived stress. Data were analyzed using structural equation modeling (SEM) and analysis of variance (ANOVA) with *post hoc* tests.

**Results:**

The results show that Frequent gardening (*β* = 0.225, *p* = 0.004) and perceiving it as a leisure activity (*β* = 0.209, *p* = 0.009) were associated with stronger attachment to gardening, which was linked to higher reported mental wellbeing (*β* = 0.256, *p* < 0.001) and lower perceived stress (β = 0.241, *p* < 0.001). Attachment partially mediated the relationship between gardening engagement and mental health outcomes. ANOVA indicated that participants who gardened regularly reported higher mental wellbeing than those who did not, with the most notable associations observed among individuals who gardened at least twice a week.

**Conclusion:**

Participants who garden report higher mental health and lower stress levels, especially when gardening frequently with leisure motivation and a sense of attachment. To create healthier cities, public health and urban planning initiatives should promote access to leisure gardening activities and develop future green gardening tourism initiatives.

## 1 Introduction

Humans are biopsychosocial beings inextricably linked to nature (White et al., 2023). However, modern urbanization, digitalization, and technological progress have increasingly separated people from their natural environments (Kurilenko et al., 2022). These civilizational processes contribute to economic growth and improved service quality but also to mental health problems. Nature deficit syndrome refers to psychological issues that arise from insufficient exposure to nature (Wang et al., 2023). These include Internet addiction, cyber sickness, and digital fatigue (Ng et al., 2025).

Living in isolation from nature, many people lose a valuable resource for mental health restoration (Willis et al., 2024). The human-environment system is becoming increasingly complex as artificial environments replace natural ones (Gaston et al., 2023). Interaction with nature is becoming secondary for most city dwellers, and even proponents of digitalization acknowledge its negative consequences. As reported by Souchet et al. (2023), prolonged use of video interfaces can lead to mental fatigue in users. This awareness has increased interest in pro-ecological thinking, nature-based interventions and environmental protection (Liang et al., 2023; Mamirkulova et al., 2025; Mamirkulova et al., 2020).

Understanding how natural interactions affect human psychological wellbeing has become increasingly urgent (Shrestha et al., 2025). Gardening is one of the most accessible forms of nature-based ecotherapy. It is a psychological approach that uses contact with nature to address physical and mental health problems (Summers and Vivian, 2018). Unlike passive activities such as walking in a park, gardening actively engages people with plants (Nartova-Bochaver and Mukhortova, 2020). It involves growing and caring for plants for consumption and aesthetic pleasure (Lucatero and Fairbairn, 2025). A considerable body of research has examined the relationship between gardening and mental health and wellbeing in developed nations (Howarth et al., 2020; Wang and Boros, 2025). However, few scholars have explored the relationship between gardening and mental health and wellbeing in developing countries. Gardening and green areas are differentiated from developed countries because of accessibility, availability, and perception among gardeners. Similarly, Ainamani et al. (2022) called for an exploration of the mental health benefits of gardening in low- and middle-income countries (LMICs). Therefore, the present study extends their research to the urban context of Shymkent in Kazakhstan. We focus on private gardening (dacha and homestead), an everyday livelihood activity in a metropolis (Mamirkulova et al., 2020). Kazakhstan’s socioeconomic diversity serves as a key case study. Gardening intersects with cultural and culinary practices. From a geographical context, we address the limitations given by Ainamani et al. (2022). Kazakhstan’s national policies, such as the “Roadmap for the Development of Dacha and Gardening Farms for 2024–2026” (Zakon.kz, 2023), politically support gardening as a leisure and agro activity. Government policy has made empirical research an urgent priority.

There is a well-recognized relationship between gardening and health and wellbeing; the next focus to be addressed is how these connections are created. Studies have shown that activity in green spaces can have both direct and indirect benefits on wellbeing (Ainamani et al., 2022; Panțiru et al., 2024; Zhao et al., 2024). These effects can be attributed to their frequency of use and the sense of leisure associated with these activities. In contrast, frequency indicates how often a person enters public areas for various purposes over a specific period. Research has shown that people’s physical and mental health are favorably correlated with frequent visits to public areas (Chalmin-Pui et al., 2021; Hong et al., 2019). Numerous studies have shown that having a green mindset has benefits, even in the absence of actual use (van den Berg et al., 2003).

Given the compelling evidence for the benefits of gardening, a key question arises: Why do some people not share a fondness for gardening? At the very least, a key explanation lies in their personal history. For many, the first impression of gardening is associated with it being a mandatory duty. This creates negative associations that inhibit its adoption as a voluntary and pleasurable pursuit. In contrast, the literature recognizes that for the therapeutic benefits of gardening, it must be experienced as a leisure activity (Fox, 2017). Hence, gardening is viewed as a leisure activity with a mindset of no obligation, referring to an internally motivating and pleasing activity. Above all, the impact of gardening on wellbeing is not direct but can be mediated by the development of an emotional attachment to the activity. The concept of forming a restorative bond with nature is supported by environmental psychology theories (Kaplan, 1987; van den Berg et al., 2003).

**Figure 1:**
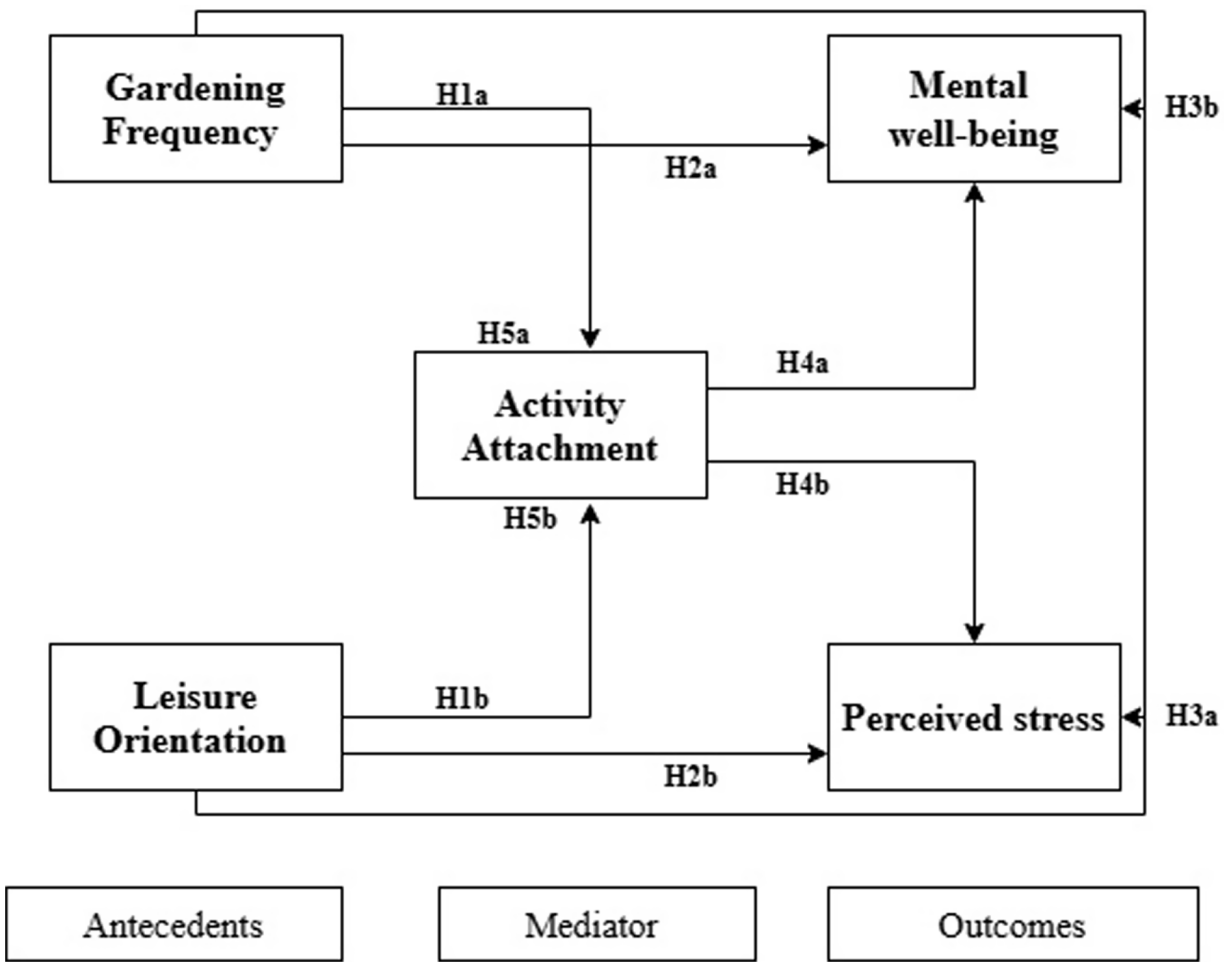
Conceptual framework.

Hence, our study aims to examine how gardening frequency and leisure orientation contribute to activity attachment, as they establish a profound psychological connection with gardening practice (See Figure 1). In turn, activity attachment mediates improvements in mental wellbeing and stress reduction. Our study findings aim to provide both theoretical and practical implications. These findings can guide local health and urban planning policies for developing future natural resource-based wellness initiatives, such as horticultural tourism, in developing countries. Our study theoretically extends attachment theory from interpersonal and place contexts to the domain of activity attachment in nature-based leisure. This was achieved by combining concepts from leisure studies with environmental psychology, demonstrating that intrinsic motivation is a significant mechanism in nature-related activities.

The conceptual framework in Figure 1 proposes that gardening frequency and leisure orientation have a (1) direct effect on mental wellbeing and perceived stress and (2) an indirect effect mediated through activity attachment.

## 2 Literature review and hypotheses operationalization

### 2.1 Theoretical framework

Based on attachment theory, this study builds a theoretical framework. This theory explains the psychological bonds people have with people, places, and activities (Moore, 2002; Scannell and Gifford, 2010; Shen et al., 2022; Tsaur et al., 2014). This theory has been successfully extended to human interactions with the environment (Barrable, 2025). Barrable (2025) explains how an emotional connection with nature helps to strengthen feelings of security, belonging, and identity. There is an essential distinction between gardening as a pastime and as an obligation. Research on psychological stress has confirmed that personal responsibility is a significant stressor (Rink et al., 2023). High personal responsibility for task completion results directly in psychological tension and physiological stress reactions (Iwanaga et al., 2000). It shows that high-stakes situations with great responsibility can be discouraging. In our research model, we assumed that perceiving gardening as a voluntary leisure activity rather than a mandatory task determines its psychological impact. Leisure orientation fosters positive attachment through repeated and meaningful engagement, enhancing personal satisfaction and wellbeing (Shen et al., 2022; Valencia et al., 2022). According to attachment theory, connections with safe and comforting objects provide a sense of security and basic comfort, which reduces stress (Shen et al., 2022; Valencia et al., 2022). When people develop a strong attachment to gardening through a leisure-oriented approach, it becomes an integral part of their identity (Kiesling and Manning, 2010) and coping repertoire (Fjaestad et al., 2023). The immersive and mindful nature of gardening requires focused attention on the present moment (Macaulay et al., 2022). This helps break the cycles of thought that are central to stress and anxiety (Shen et al., 2023). Thus, we constructed a conceptual model that includes both behavioral engagement (frequency) and psychological orientation (attitude toward leisure), which predicts attachment to the activity. As a result, attachment to the activity mediates the relationship between these antecedents and improvements in mental wellbeing, as well as reductions in stress. These findings have implications for the future development of horticultural ecotourism. The basic idea of horticultural ecotourism programs is that they serve as low-commitment and easily accessible entry points. It promotes a leisure mindset, which gives them special significance (Fox, 2017). Visitors participating in ecotourism can enjoy gardening without bearing full responsibility for the long-term care of the garden or the harvest. Being a “guest” is a psychological trait that relieves the pressure associated with performance (Jordan et al., 2019). It turns the activity into pure leisure and is perfect for fostering attachment to it, which has a positive impact on mental health (AlAli et al., 2024). Thus, studying gardening is aimed at optimizing psychological outcomes through the promotion of a leisure-oriented approach to gardening, which is relevant to developing future tourism products.

### 2.2 Hypothesis operationalization

A high frequency of visits to green areas and private gardens positively impacts physical health and the prevention of chronic diseases (Chalmin-Pui et al., 2021; Hong et al., 2019). Chalmin-Pui et al. (2021) reported that gardening at least 2–3 times a weekly is associated with the most significant health benefits. This includes enhanced physical activity levels and improved overall wellbeing. Garden settings provide fresh air exposure, suppress pathogens, enhance immune responses, and positively influence various physiological systems (Andersen et al., 2021). The importance of garden spaces has become particularly apparent during the COVID-19 pandemic. For example, Corley et al. (2021) found that home gardens served as core health resources for older adults during the lockdown. We propose that repetitive behavioral engagement directly contributes to attachment to an activity.

In line with attachment theory, recent studies suggest that attachment may develop not only toward places but also toward recurrent, meaningful activities embedded in everyday life, such as gardening. Prior research has shown that the core dimensions of attachment theory positively and significantly influence individuals’ pro-environmental behavior and overall wellbeing (Shen et al., 2022). Building on this theoretical foundation, the present research focuses on one attachment dimension, activity attachment, within the context of gardening. Gardening is a long-term, place-based, and practice-oriented activity. It involves repeated interactions with natural elements, making it particularly conducive to the development of emotional bonds through sustained engagement. In this context, activity attachment refers to the extent to which individuals feel emotionally involved and connected to gardening-related practices and experiences. These activities may occur in private or community gardens and include planting, cultivation, and harvesting. These include other routine practices that collectively shape a broader gardening experience (Hume et al., 2022). By emphasizing activity attachment, this study extends existing attachment research and highlights gardening as a distinctive form of activity through which psychological connections and wellbeing can emerge. For example, research in the physical activity domain suggests that individuals’ attachment orientations significantly shape how and why they engage in sustained activities, including their preferences for activity type, intensity, and social context (Hill et al., 2024). This study offers relevant insights into the engagement patterns in gardening. Regular engagement enables individuals to develop familiarity with the activity, skills, and emotional connections associated with it (Shen et al., 2022; Tsaur et al., 2014). Our assumptions also align with the recreation specialization theory, which suggests that greater engagement in leisure activities strengthens place identity and place dependence (Huo et al., 2025).

Hypothesis 1a (*H1a*): Higher gardening frequency is positively associated with stronger activity attachment.

In high-income countries, gardening is often a leisure activity, whereas in low-income countries, it is a means of providing a livelihood for the family. For instance, many people in Australia and England enjoy gardening or flower cultivation as peaceful and satisfying hobbies (Cheng and Pegg, 2016; Fox, 2017). Alaimo et al. (2024) found that engagement in gardening among new gardeners in the USA resulted in nearly universal benefits, including food production, physical activity, and a core “gardening triad” of psychological rewards, such as the nurturance of plants, feelings of responsibility, and a restorative connection to nature. However, a realist review by Flores et al. (2025) clarifies that motivation is highly context-dependent and that the path to these advantages is not foreseeable. Food security, curiosity, and self-efficacy are essential motivators (Flores et al., 2025). In addition to individual motivations, gardening as a leisure-oriented activity also carries important social and experiential meanings. Empirical evidence shows that time spent in gardens and a stronger connection to nature are associated with higher levels of social cohesion, trust, and interaction, particularly in urban contexts (Oh et al., 2022). These findings suggest that leisure gardening facilitates personal wellbeing and socially embedded experiences. Similarly, research indicates that individuals’ motivations and preferences are strongly shaped by the characteristics of the activities they engage in, and that voluntary and enjoyable participation is essential for generating positive experiences (Valencia et al., 2022). In gardening, engaging in these practices by choice can enhance enjoyment, meaning, and emotional involvement. Together, these studies support the view that when gardening is approached as a leisure activity rather than an obligation, it is more likely to foster meaningful engagement and attachment. In contrast, a behavioral study by Iwanaga et al. (2000) suggests that a sense of high responsibility for one’s task enhances psychological responses. Therefore, the benefits of gardening are most effectively realized when this activity is perceived as an intrinsically motivated leisure pursuit rather than obligatory work (Rubel et al., 2024). Attachment theory suggests that the voluntary and enjoyable nature of leisure-oriented approaches promotes a strong attachment to activities (Shen et al., 2022). Thus, we hypothesized that a psychological orientation toward gardening is essential.

Hypothesis 1b (*H1b*): Higher leisure orientation toward gardening is positively associated with stronger activity attachment.

#### 2.2.1 Direct effects of antecedents on mental health outcomes

Research has demonstrated that frequent gardeners report higher levels of happiness and life satisfaction than those who garden irregularly (Fjaestad et al., 2023). Regular physical activity promotes endorphin release and cortisol reduction, and repeated exposure to natural environments provides restorative benefits (Chalmin-Pui et al., 2021; Macaulay et al., 2022). Individuals who garden at least twice a week have significantly lower stress levels (Koay and Dillon, 2020). Over 50 percent of the 433 older adults who participated in a gardening activity in Australia reported that their leisure, mental health, and physical wellbeing increased significantly (Cheng et al., 2010). A leisure mindset alleviates performance pressure and fosters conditions for relaxation and enjoyment (Iwanaga et al., 2000). This leads to immediate mood enhancement and reductions in cortisol levels and self-reported stress (Murtagh and Frost, 2023).

Hypothesis 2a (*H2a*): Gardening frequency is positively associated with mental wellbeing.

Hypothesis 3a (*H3a*): Gardening frequency reduces perceived stress.

Hypothesis 2b (*H2b*): Leisure orientation increases mental wellbeing of the elderly.

Hypothesis 3b (*H3b*): Leisure orientation reduces perceived stress.

The positive impact of gardening on mental health is well recognized (Andzaurova and Nartova-Bochaver, 2023; Panțiru et al., 2024). Horticultural therapy helps to reduce anxiety and depression, as well as help to improve concentration and self-esteem (Allison et al., 2024; Andzaurova and Nartova-Bochaver, 2023). Activity attachment is a psychological bond formed through engagement. It is proposed that this will have an impact on sustained mental health benefits. For example, a study of women participating in nature-based leisure showed that physical experiences, including sight, smell, taste, and movement (Ridgway, 2024), facilitated emotional and affective connections with nature. According to attachment theory, bonds with secure and restorative entities provide a sense of safety and comfort during stress recovery (Shen et al., 2022; Valencia et al., 2022). When such a bond is formed through gardening, the practice is transformed from a simple task into a reliable personal resource for meeting core psychological needs for meaning, mastery, and restoration. Hence, it is proposed:

Hypothesis 4a (*H4a*): Activity attachment increases mental health.

Hypothesis 4b (*H4b*): Activity attachment reduces the perceived stress.

Hypothesis 5a (*H5a*): Activity attachment mediates the relationship between gardening frequency, mental wellbeing, and perceived stress.

Hypothesis 5b (*H5b*): Activity attachment mediates the relationship between leisure orientation, mental wellbeing, and perceived stress.

## 3 Methodology

### 3.1 Study locale

The study was conducted in Shymkent, the third-largest city in Kazakhstan. It has a population of approximately 1.2 million. There are three reasons for selecting Shymkent. First, the city became a third metropolis in 2018. Second, despite rapid urbanization, Shymkent maintains close cultural ties to traditional dacha gardening methods entrenched in Soviet traditions (Yussupova et al., 2019). This creates a unique context for examining the psychological mechanisms of gardening. Third, the study coincides with Kazakhstan’s “Roadmap for Development of Dacha and Gardening Farms for 2024–2026” (Zakon.kz, 2023), which aims to transform private garden plots into accessible green spaces and enhance community wellbeing. However, this policy lacks localized empirical evidence. Our research addresses this gap by examining the psychological impacts of gardening among urban residents in various forms, such as with indoor plants, in private yards, on community plots, or at suburban dachas.

### 3.2 Research design

This study used a quantitative cross-sectional design to test a complex mediation model examining the relationships between gardening frequency, leisure orientation, activity attachment, and psychological wellbeing outcomes. The cross-sectional design of the study allowed us to capture current gardening practices and psychological states among a diverse urban population at a single point in time. Subsequently, we demonstrated the relationships between the variables. The design also allowed for a comparison of the results between gardeners and non-gardeners, as well as an examination of the variability within the gardener population. Structural equation modeling (SEM) was selected as the primary analytical approach. It helps in the simultaneous testing of multiple direct and indirect paths, provides an overall assessment of model fit, and accounts for measurement error in latent constructs.

### 3.3 Ethical considerations

We obtained ethical approval from the Shandong Xiehe University Ethics Committee (approval number: LLSC-KY03-2025028). Data were collected between March and April 2024. All procedures conformed to the Declaration of Helsinki (1964), and the participants provided informed consent before participation.

### 3.4 Sampling strategy

The non-probability sampling snowball method was employed. This method is essential because Shymkent lacks a comprehensive sample of urban gardeners. The author’s personal experience with the region’s dacha culture provided access to local connections and an understanding of a culturally sensitive approach to research. To ensure diversity among representatives, we collected data by phone and in person from owners of gardening dachas and local agricultural institutions, all of whom were aged above 18 years. The first contacted respondents were asked to forward the survey to those they thought might be suitable. The research assistants used brief personal storytelling techniques to explain the purpose of the study and encourage participants to participate. As noted by Mamirkulova et al. (2020) and Abbas et al. (2025), this is effective in the Central Asian cultural context. Indeed, the cultural characteristics contributed to a high response rate of approximately 70.3%. Of the 300 questionnaires sent, 210 valid responses were received for analysis.

### 3.5 Data collection instruments

For the current research, data were collected using a structured self-administered questionnaire. Following Brislin (1970), the questionnaire was first translated from English into Kazakh and Russian languages. An independent back-translation was then conducted to ensure conceptual equivalence and cultural appropriateness for the population of Shymkent, Kazakhstan. Some items were adapted to reflect the local cultural context, as direct translations could not fully capture specific meanings. For example, minor adjustments were made to the direct use of the word “dacha” when describing gardening activities. This term has exceptional cultural significance in Kazakhstan, and the words “garden” or “plot” do not fully capture it. The final questionnaire consisted of five sections: demographic information, gardening status and profile, leisure orientation, activity attachment, and outcome scales.

### 3.6 Participant screening and measurement procedure

**Figure 2:**
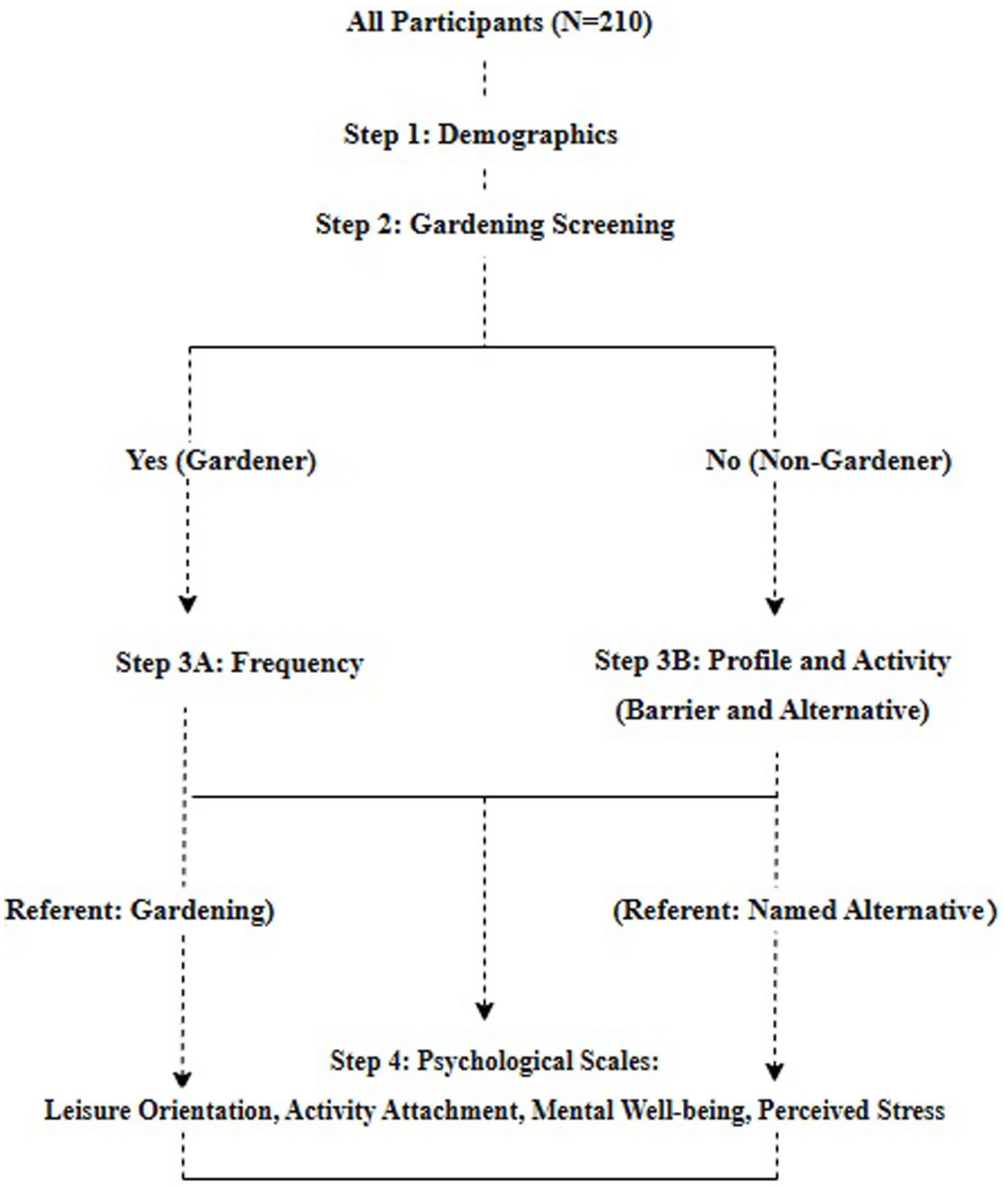
Participant screening and measurement flow.

The study used a sequential screening and measurement procedure to accurately classify participants and ensure that the measurement of all psychological constructs was appropriate (see Figure 2).

*Step 1:* Demographic Information. All participants provided standard demographic information (gender, age, education, profession, and living status).

*Step 2:* Primary Gardening Status Screening. All potential participants first answered a screening question to determine their eligibility for the study’s core focus and to establish the primary comparison groups: *“In the past 12 months, have you engaged in any gardening activities? This includes caring for indoor plants, balcony or container gardens, private yard gardens, community garden plots, or dacha plots in suburban areas.”* Respondents answering “Yes” were classified as gardeners and proceeded to Step 3A. Those answering “No” were classified as non-gardeners and proceeded to Step 3B.

*Step 3A:* Gardening Profile. Gardeners were asked to specify their engagement frequency*: “On average, how often have you engaged in gardening in the past 3 months?”* Response scale: 1 = Once per week, 2 = Twice per week, 3 = Three times per week or more.

*Step 3B:* Non-gardeners completed a two-part question: (a) selecting the main reason they do not garden from a list of barriers (e.g., lack of space, time, and skills), and (b) naming their primary alternative leisure activity in an open-ended text field (e.g., reading, watching TV).

*Step 4:* Measurement of Psychological Constructs. All participants then completed the core psychological scales, with instructions tailored to their group to provide concrete references.

*For Gardeners*, the items in the Leisure Orientation and Activity Attachment scales referred specifically to their gardening activity.

*For Non-Gardeners*, the same scales referred to the specific alternative activity they named in Step 3 B.

*The following scales were administered:*

Leisure Orientation: A 4-item scale adapted from Fox (2017). Participants rated their agreement with statements such as *“I approach this activity primarily as a form of leisure and relaxation,”* on a 7-point Likert scale (1 = Strongly Disagree to 7 = Strongly Agree).

Activity Attachment: A 5-item scale adapted from Shen et al. (2022). Participants rated statements such as “This activity brings me good memories” on the same 7-point Likert scale.

Mental wellbeing: Measured using seven items adapted from the WHO-5 wellbeing index (de Wit et al., 2007) on a 7-point Likert scale.

Perceived Stress: measured using an adapted 10-item perceived stress scale (PSS) from Cohen et al. (1983) on a 7-point frequency scale (1 = never to 7 = very often). Negatively worded items were reverse-coded, such that higher final scores indicated lower perceived stress. All the scale items are listed in Table 1.

**Table 1:** Scales of the used questionnaire.

Scale	Codes	Items
Leisure orientation	LO1	I approach this activity primarily as a form of leisure and relaxation
	LO2	For me, this activity is an escape from daily responsibilities
	LO3	I feel no pressure to achieve perfect results in this activity
	LO4	I participate in this activity because I enjoy the process and, not just the outcome.
Attachment activity	AA1	I like this activity very much.
	AA2	This activity brings me good memories.
	AA3	This activity attracts me more than other leisure activities.
	AA4	This activity is exciting to me.
	AA5	The experience of doing this activity makes me appreciate related things more.
Mental wellbeing	MWB1	I have felt cheerful and in good spirits.
	MWB2	I have felt calm and relaxed.
	MWB3	I have felt active and vigorous.
	MWB4	I woke up feeling fresh and rested.
	MWB5	My daily life has been filled with things that interest me.
Perceived stress	PS1	How often have you been upset because of something that happened unexpectedly in the last month?
	PS2	How often have you felt that you were unable to control the important things in your life in the last month?
	PS3	How often have you felt nervous and stressed in the last month?
	PS4	How often have you felt confident about your ability to handle your personal problems in the last month?
	PS5	How often have you felt that things were going your way in the last month?
	PS6	How often have you found that you could not cope with all the things that you had to do in the last month?
	PS7	How often have you been able to control irritations in your life in the last month?
	PS8	How often have you felt that you were on top of things in the last month?
	PS9	How often have you been angered because of things that happened that were outside of your control in the last month?
	PS10	How often have you felt difficulties were piling up so high that you could not overcome them in the last month?

### 3.7 Validation process

Five academic experts were invited to review the initial version of the questionnaire. Their comments helped us refine the question wording and scale structure and adjust for cultural specifics in Kazakhstan. Next, a pilot test was conducted with a small group (*n* = 20) that was representative of the target population. After official data collection (*n* = 210) was completed, the internal consistency of all multi-item scales was assessed using Cronbach’s alpha, and all scales were above 0.75. The results confirmed the reliability of the measurements in the study sample.

### 3.8 Data analysis

The collected data were analyzed using a two-stage analytical approach with IBM SPSS Statistics Version and IBM SPSS AMOS.

#### 3.8.1 Stage 1: preliminary analyses

Descriptive statistics were used to characterize the sample. Cronbach’s alpha confirmed the internal consistency of all multi-item scales. Principal component analysis with Promax rotation established the measurement model structure, and KMO and Bartlett’s test confirmed data suitability. Common method bias was assessed using Harman’s single-factor test and the marker variable technique, with no single factor explaining most of the variance.

#### 3.8.2 Stage 2: hypothesis testing

Confirmatory factor analysis (CFA) in AMOS validated the measurement models. The results of the constructs demonstrate discriminant validity via the Fornell-Larcker criterion (AVE > MSV). Structural Equation Modeling (SEM) was then used to test the theoretical model and provide an assessment of all direct and indirect effects. To examine group differences, One-Way ANOVAs with Tukey’s HSD post-hoc tests were used to compare outcomes across the four gardening frequency groups. Finally, the 2 × 2 typology of gardeners (cross-tabulation of frequency and leisure orientation) was tested using analysis of variance (ANOVA) to compare the five resulting groups across all psychological outcomes.

## 4 Results

### 4.1 Descriptive statistics

As shown in Table 2, the demographic profile of respondents (*n* = 210) shows a predominance of women (60.5%) and middle-aged and older individuals, with 61.4% of the participants aged 41 years and older. Educational attainment was distributed almost evenly, with a slight majority (55.7%) holding less than a bachelor’s degree. The occupational profile indicates that government employees and homemakers are the major participants (29.0% each). Living status revealed a strong family orientation, since 49.0% of the respondents lived with their families.

**Table 2:** Demographic profile of the study participants.

Variables	Dimensions	Frequency	Percentage
Gender	Male	83	39.5%
	Female	127	60.5%
Age	19–30	38	18.1%
	31–40	43	20.5%
	41–50	67	31.9%
	Above 51	62	29.5%
Education	Middle (lower than bachelor’s)	117	55.7%
	High (bachelor and above)	93	44.3%
Profession	Government Job	61	29.0%
	Non-Government Organization	51	24.3%
	Retired	45	21.4%
	Students	6	2.9%
	Self-employed	47	22.4%
	Housewife	61	29.0%
Living status	Living alone	18	8.6%
	Living with relatives	34	16.2%
	Living with family (e.g., spouse, parents, children)	103	49%
	Living with roommates, friends, /non-relative	55	26.2%

Table 3 presents the characteristics of the gardener and non-gardener participants in this study. Nearly two-thirds of the participants (64.3%, *n* = 135) reported engaging in gardening within the past 12 months. In particular, the most common frequency was gardening three times a week (30.5%). Among those who did not garden (35.7%, *n* = 75), the primary barriers were lack of space (24%), lack of time (20%), and lack of knowledge/skills (16%). Sixteen percent of non-gardeners did not specify reasons, and some reported motivational barriers, such as not wanting to take responsibility for the process (10.7%), a lack of interest (6.7%), or high costs (6.7%). When examining alternative leisure activities among non-gardeners, nearly half (49.3%) primarily engaged in passive activities such as watching television and movies (29.3%) or using social media and the Internet (13.3%). Active leisure activities, such as sports/physical education (16.0%) and reading (20.0%), accounted for approximately one-third of the alternative options. The distribution indicates that the leisure profiles of non-gardeners differed significantly from those of gardeners, for whom gardening was invariably the primary leisure activity.

**Table 3:** Characteristics of gardeners and non-gardeners (*N* = 210).

Gardening participation (*n* = 135)		
Gardening frequency	Frequency	Percentage
Never	75	35.7%
once a week	24	11.4%
twice a week	47	22.4%
three times a week	64	30.5%
Total gardeners	135	64.3%

Characteristics of non-gardeners (*n* = 75)		
Barriers to gardening		
Barriers	Frequency	Percentage
Lack of space (no balcony/yard)	18	24.0%
Lack of time	15	20.0%
Lack of knowledge/skills	12	16.0%
No interest	5	6.7%
Do not want to take responsibility for the entire gardening	8	10.7%
Too expensive to rent gardening land	5	6.7%
Other	12	16.0%
Total	75	100%

Most common alternative activities		
Activity	Frequency	Percentage
Watching TV/Movies	22	29.3%
Reading	15	20.0%
Sports/Exercise	12	16.0%
Social Media/Internet	10	13.3%
Socializing with friends/family	8	10.7%
Other hobbies (crafts, music, etc.)	8	10.7%
Total	75	100%

### 4.2 Factor analysis

Principal component analysis with Promax rotation was conducted to examine the factor structure of all the scale items. The Kaiser-Meyer-Olkin measure (0.870) and a significant Bartlett’s Test of Sphericity (*p* < 0.001) confirmed the excellent suitability of the data for factor analysis. The analysis revealed a factor that accounted for 66.68% of the total variance. Moreover, the pattern matrix revealed that all items loaded above 0.70 on their intended factors, without significant cross-loadings. However, we removed one item of mental wellbeing (MWB5) and one item of activity attachment (AA5), as well as four items of perceived stress (PS4, PS5, PS7, PS8) due to a loading factor below 0.70 in the factor analysis. High communalities across the remaining items indicated that the retained items were well explained by the extracted factors. In addition, it provides confirmation of scale validity and reliability.

### 4.3 Common method bias

The author applied Harman’s one-factor test to assess the common method bias (CMB). This test revealed eight factors that cumulatively accounted for 36.12% of the variance, which was below the 50% threshold. This confirmed that the CMB was not an issue in our data. Furthermore, it was supported by a simple marker variable method, which also confirmed the minimal standard method variance (Simmering et al., 2015). No significant demographic differences were observed (*p* > 0.05).

### 4.4 Reliability and validity tests

All scales demonstrated excellent reliability (Cronbach’s *α* ranging from 0.843 to 0.948), well exceeding the 0.70 threshold. Respondents reported a high average level across all constructs (means 5.45–5.69 on 7-point scales), with a significant negative skew (skewness −1.09 to −1.56). This indicates that the distributions are concentrated toward the positive ends of the scales (see Table 4).

**Table 4:** Reliability and validity test.

Scale	Codes	Factor loadings	Mean	SD	Skewness	Kurtosis	Cronbach’s α
Leisure orientation	LO1	0.912	5.66	1.778	−1.560	1.354	0.948
	LO2	0.888	5.69	1.600	−1.505	1.271	
	LO3	0.876	5.70	1.657	−1.620	1.747	
	LO4	0.897	5.64	1.658	−1.568	1.522	
Activity attachment	AA1	0.758	5.44	1.631	−1.018	0.569	0.843
	AA2	0.711	5.35	1.568	−0.945	0.595	
	AA3	0.754	5.47	1.516	−1.017	0.715	
	AA4	0.807	5.52	1.557	−1.354	1.629	
Mental wellbeing	MWB1	0.858	5.52	1.756	−1.477	1.200	0.904
	MWB2	0.858	5.60	1.620	−1.310	0.686	
	MWB3	0.831	5.61	1.753	−1.477	1.119	
	MWB4	0.766	5.59	1.670	−1.124	0.469	
Perceived stress	PS1	0.826	5.72	1.507	−1.682	2.238	0.946
	PS2	0.820	5.90	1.387	−1.609	2.059	
	PS3	0.755	5.74	1.425	−1.292	0.847	
	PS6	0.767	5.61	1.544	−1.240	0.702	
	PS9	0.712	5.51	1.541	−1.047	0.203	
	PS10	0.771	5.66	1.463	−1.295	1.044	

The composite reliability (CR) values ranged from 0.844 to 0.940, and all average variance extracted (AVE) values exceeded 0.50, supporting convergent validity. Items within each scale measure the same underlying construct (Nunnally and Bernstein, 1994). Discriminant validity was confirmed using the Fornell–Larker standard. Relatively, the square root of each construct’s AVE (diagonal values) was greater than its correlations with all other constructs (off-diagonal values), and the MSV was lower than the AVE (see Table 5).

**Table 5:** Discriminant validity.

Construct	CR	AVE	MSV	MaxR(H)	LO	AA	MWB	PS
LO	0.940	0.798	0.131	0.941	**0.893**			
AA	0.844	0.575	0.321	0.848	0.307	**0.758**		
MWB	0.898	0.687	0.117	0.902	0.214	0.342	**0.829**	
PS	0.901	0.602	0.321	0.904	0.362	0.567	0.277	**0.776**

LO: Leisure Orientation; AA: Activity Attachment; MWB: Mental wellbeing; PS: Perceived Stress. Diagonal values in bold represent the square root of AVE for each construct, used to assess discriminant validity. Off-diagonal values represent correlations between constructs.

The measurement model also demonstrated a good fit: χ^2^ = 185.187; degree of freedom = 129, TLI = 0.972; CFI = 0.977; RMSEA = 0.046. These results collectively demonstrate that the measurement model possesses adequate reliability, convergent validity and discriminant validity. The final structural equation model demonstrated an excellent fit with the data: χ^2^(146) = 222.14, *p* < 0.001, CFI = 0.969, RMSEA = 0.050 (90% CI: 0.036–0.063), GFI = 0.901, and NFI = 0.915. All fit indices exceeded the commonly recommended thresholds, with CFI and TLI values of 0.90 or above and RMSEA values below 0.08, indicating strong support for the theoretical model (Hair et al., 2019; Hu and Bentler, 1999) (see Table 6).

**Table 6:** Model fitness.

Statistical test	X2	Degree of freedom	CMIN/DF	TLI	CFI	IFI	RMSEA
Measurement model fitness CFA	185.187	129	1.436	0.972	0.977	0.977	0.046
SEM model fit	222.137	146	1.521	0.964	0.969	0.969	0.050

### 4.5 Hypothesis testing results

Structural equation modeling (SEM) analysis revealed a comprehensive network of significant relationships supporting the proposed model. All proposed paths were statistically significant (*p* < 0.05) (see Table 7).

**Table 7:** SEM results.

Path of direct effect		Estimate	S.E.	C.R.	*p-*value
H1a	Gardening frequency →Activity Attachment	0.225	0.078	2.891	0.004
H1b	Leisure Orientation →Activity Attachment	0.209	0.060	2.622	0.009
H2a	Activity Attachment →Mental wellbeing	0.256	0.096	3.335	***
H2b	Activity Attachment →Perceived stress	0.241	0.089	3.510	***
H3a	Gardening frequency →Mental wellbeing	0.226	0.089	3.151	0.002
H3b	Gardening frequency →Perceived stress	0.378	0.084	5.888	***
H4a	Leisure Orientation →Mental wellbeing	0.193	0.068	2.554	0.011
H4b	Leisure Orientation →Perceived stress	0.217	0.063	3.333	***

Path of indirect effect		Estimate	Total effect	% Mediated	Results
H5a	Gardening frequency →Activity Attachment →Mental wellbeing	0.057	0.283	(0.057/0.283) × 100 = 20.14%	Partial Mediation
	Gardening frequency →Activity Attachment →Perceived stress	0.054	0.432	(0.054/0.432) × 100 = 12.50%	Partial Mediation
H5b	Leisure Orientation →Activity Attachment →Mental wellbeing	0.053	0.247	(0.053/0.247) × 100 = 21.46%	Partial Mediation
	Leisure Orientation →Activity Attachment →Perceived stress	0.050	0.268	(0.050/0.268) × 100 = 18.66%	Partial Mediation

All effects are standardized. *p* < 0.05. For Perceived Stres**s:** A positive coefficient indicates a stress-reducing effect due to the scale being reverse-coded (higher score = lower stress). The “% Mediated” represents the proportion of the total effect that is transmitted through the mediator (activity attachment). The *** in the table as actual numbers (e.g., 0.000 or <0.001).

Direct Effects of Activity Attachment: Both gardening frequency (*β* = 0.225, *p* = 0.004) and leisure orientation (*β* = 0.209, *p* = 0.009) were significant positive predictors of activity attachment. The analysis supports H1a and H1b and indicates that more frequent engagement and a leisure-oriented mindset each contribute independently to forming a stronger emotional bond with the activity.

Direct Outcomes of Activity Attachment: Activity attachment was a significant mediator and positively predicted both mental wellbeing (*β* = 0.256, *p* < 0.001) and lower perceived stress (*β* = 0.241, *p* < 0.001). Hence, H2a and H2b support the suggestion that a stronger bond with gardening is directly associated with higher wellbeing and lower stress levels.

Direct effects of past circumstances on mental health outcomes: The frequency of gardening had a significant positive direct effect on mental wellbeing (*β* = 0.226, *p* = 0.002) and perceived stress (*β* = 0.378, *p* < 0.001), independent of activity attachment. Hence, H3a and H3b are supported. Similarly, leisure orientation had a significant positive direct effect on mental wellbeing (*β* = 0.193, *p* = 0.011) and perceived stress (*β* = 0.217, *p* < 0.001), independent of activity attachment. Therefore, the results support H4a and H4b.

Mediating Role of Activity Attachment: The analysis confirmed activity attachment as a significant partial mediator for all paths (see Table 8). Significant indirect effects were found for gardening frequency on wellbeing (*β* = 0.057, 20.1% of total effect) and stress (*β* = 0.054, 12.5%), supporting H5a. In addition, for leisure orientation on wellbeing (*β* = 0.053, 21.5%) and stress (*β* = 0.050, 18.7%), supporting H5b.

**Table 8:** ANOVA results and descriptive statistics by gardening frequency.

Outcome variable	Group	*N*	Mean	SD	*F*(df = 3,206)	*p*-value	η^2^	*Post-hoc* results
Activity	Never	75	4.90	1.69	7.60	<0.001	0.10	Never < Once/week, Twice/week, Three/week
	Once a week	24	5.73	0.89				(No diff. Among gardeners)
	Twice a week	64	5.75	0.88				
	Three times a week	47	5.75	0.87				
Wellbeing	Never	75	4.82	2.04	10.70	<0.001	0.13	Never < Twice/week, Three/week
	Once a week	24	5.44	1.42				(Once/week = Never, ns)
	Twice a week	64	6.07	0.72				(No diff. Among gardeners)
	Three times a week	47	5.98	0.87				
Stress	Never	75	4.18	2.02	33.85	<0.001	0.33	Never < Once, Twice, Three/week
(1 = High, 7 = Low)	Once a week	24	5.92	0.63				(No diff. Among gardeners)
	Twice a week	64	6.11	0.42				
	Three times a week	47	6.02	0.69				

NS = not significant, η^2^ = partial eta squared (effect size: 0.01 = small, 0.06 = medium, 0.14 = large). Perceived stress was reverse-coded; a higher score indicates lower stress.

#### 4.5.1 Dual-pathway model interpretation

These results support a dual-pathway model for the psychological benefits of gardening. The first pathway consists of strong direct effects of behavioral engagement (frequency) and psychological orientation (leisure) on outcomes. The second pathway is the attachment-mediated pathway. These antecedents promote attachment to the activity, which, in turn, improves wellbeing and reduces stress.

The larger magnitude of the direct effects, particularly from gardening frequency to reduced stress (*β* = 0.378), suggests that the immediate benefits of physical engagement and leisure respite are dominant. The significant indirect effects demonstrate that the attachment-mediated pathway provides a meaningful supplementary mechanism. Since it accounts for 12–22% of the total effects, it captures the resilient psychological integration of gardening as a valued and identity-relevant resource for older adults.

### 4.6 ANOVA results of gardening participation and non-participation

To assess the impact of gardening frequency on psychological outcomes, a series of one-way ANOVAs was performed. It compared four groups that involved non-gardeners and those who gardened once, twice, or three times per week. The analysis found that the most significant factor determining psychological benefits was whether a person gardened at all (See Table 8).

As shown in Table 8, statistically significant differences were observed between the gardening frequency groups for all three outcome variables. *Post-hoc* Tukey HSD tests revealed three key outcomes, which are shown below. A large and significant effect was found for perceived stress (η^2^ = 0.33). The non-gardener group reported significantly higher stress levels than all the gardening groups (all *p* < 0.001). In particular, there were no significant differences in stress among the groups that gardened once, twice, or thrice a week. These results indicate that any regular gardening engagement, regardless of frequency, offers substantial stress-reduction benefits. This accounted for approximately 33% of the variance in perceived stress. A different correlation was found for mental wellbeing (η^2^ = 0.13). The Non-Gardener group had significantly lower wellbeing than the twice-a-week (*p* < 0.001) and thrice-a-week (*p* < 0.001) groups. However, it was not substantially different from the once-weekly group (*p* = 0.263). The results suggest a frequency effect, which suggests that to improve mental wellbeing significantly, gardening should be done twice a week or more often compared to people who do not garden. For activity attachment (η^2^ = 0.10), there was a tendency towards lower stress. The non-gardener group reported significantly lower attachment than all the gardening groups (all *p* < 0.05), with no differences among the gardening frequency groups. The results suggest that regardless of how often one gardens, a psychological bond with gardening develops through engagement.

#### 4.6.1 Gardener typology

Gardener typology was developed using a 2 × 2 framework based on gardening frequency (high and low) and leisure orientation (high and low) (see Table 9). The categories were informed by empirical data from our survey and ideas from previous literature on leisure and recreation specialization (Huo et al., 2025; Tsaur et al., 2014). This highlights how differences in engagement and psychological orientation shape activity attachment and wellbeing outcomes. Specifically, high and low frequency was determined from participants’ self-reported gardening visits, and high and low leisure orientation was defined based on survey responses regarding their perceived voluntariness and enjoyment of gardening. This approach helps differentiate gardener groups and allows us to examine psychological outcomes.

**Table 9:** Gardener typology of descriptive statistics and group comparisons.

Gardener type	*N*	Frequency and leisure profile	Activity attachment	Mental wellbeing	Perceived stress
GT	*N*	*F*	*M* (SD)	*M* (SD)	*M* (SD)
Non-gardener	75	Control group	4.90 (1.69)	4.82 (2.04)	4.18 (2.02)
Active gardeners	68	High frequency, high leisure	5.78 (0.83)	6.01 (0.93)	6.13 (0.46)
Dutiful gardener	43	High frequency, low leisure	5.70 (0.95)	6.06 (0.49)	5.99 (0.67)
Reluctant gardener	11	Low frequency, low leisure	5.88 (0.71)	5.25 (1.61)	6.05 (0.68)
Casual enjoyer	13	Low frequency, high leisure	5.55 (0.95)	5.66 (0.82)	5.77 (0.55)

Gardener types reflect a combination of self-reported frequency, leisure orientation scores, and established literature on activity engagement.

One-way ANOVAs comparing these five groups were significant for all outcomes (all *p* < 0.001). However, post-hoc tests revealed that the divide remained between non-gardeners and all gardener types (see Tables 9, 10). For perceived stress, the outcome with the largest effect size, the non-gardener group was significantly different from all four gardener types (all *p* < 0.01). At the same time, there were no significant differences among any of the gardener types themselves (all *p* > 0.05). A nearly identical correlation was found for the activity attachment. For mental wellbeing, non-gardeners were significantly lower than the two high-frequency gardener types (Active Gardeners and Dutiful Gardeners, both *p* < 0.001).

**Table 10:** Psychological outcomes by gardener typology.

Label as gardener typology	Low gardening frequency	High gardening frequency
High leisure orientation	CASUAL ENJOYERS (G4) *** High Attachment *** Low Stress ** Moderate wellbeing	ACTIVE GARDENERS (G1) (winner) *** Highest Attachment *** Lowest Stress *** Highest wellbeing
Low leisure orientation	RELUCTANT GARDENERS (G3) ** Medium attachment *** Low Stress * Low wellbeing	DUTIFUL GARDENERS (G2) *** High Attachment *** Low Stress *** Highest wellbeing

* = Poor, ** = Moderate, *** = Good.

## 5 Discussion

This study aimed to uncover the psychological mechanisms underlying the established correlations between gardening and wellbeing. By testing a comprehensive mediation model in Shymkent, Kazakhstan, our findings offer significant enhancements to existing models of nature-based wellbeing and provide actionable insights for policy makers. The most reliable conclusion, confirmed by the analysis of variance results, was the sharp contrast between gardeners and non-gardeners, which is similar to the findings of Koay and Dillon (2020). Non-gardeners reported significantly higher stress and lower wellbeing and activity attachment than all gardener groups. Importantly, no statistically significant differences were observed among the gardeners. The results support a two-group model, where the primary driver of psychological outcomes is the simple distinction between engaging in gardening and not engaging in gardening. These findings are similar to those of a meta-analytic study by Panțiru et al. (2024), which showed the positive role of horticultural therapy and gardening in improving health, wellbeing, and general quality of life among general and vulnerable subgroups.

Moreover, SEM revealed that gardening frequency and leisure orientation positively predicted higher activity attachment. This means that when people garden more than once a week as a hobby or enjoy it, they form a strong attachment to the gardening activity. Strong activity attachment was a significant mediator that positively increased mental wellbeing and decreased perceived stress. The results also support the attachment theory propositions that bonds with restorative activities provide safety and comfort (Shen et al., 2022; Valencia et al., 2022). When people develop a strong attachment to gardening, participation becomes more than just a hobby. It becomes fully integrated into one’s personal identity and arsenal of coping strategies (Kiesling and Manning, 2010).

These findings extend the literature on recreation specialization (Huo et al., 2025). Based on their study, we suggest that as people become more specialized in gardening, they develop a stronger attachment, which leads to a significant improvement in their wellbeing. The process of specialization is similar to the place attachment mechanisms. In other words, emotional connections with the natural environment are associated with improved psychological functioning (Barrable, 2025). Additionally, the SEM results confirmed two different pathways to mental wellbeing and stress reduction. The first is a direct path, in which gardening frequency and leisure orientation lead to immediate benefits (Andersen et al., 2021; Chalmin-Pui et al., 2021; Glazener et al., 2021). The second is the indirect path via attachment. The same antecedents increase activity attachment, which enhances outcomes. This implies that the significant difference between a gardener and a non-gardener is explained by the more significant direct benefits of participation. Simultaneously, the attachment path provides a significant additional reinforcement effect for those engaged in leisure-oriented gardening.

Our findings suggest that gardening has a frequency effect. This means that any activity is helpful, but more frequent engagement leads to higher health benefits. Gardening even once a week provides significant benefits to wellbeing, such as stress reduction, compared with not gardening. However, participants who gardened two or three times weekly reported substantially higher overall wellbeing than those who gardened once. For example, scholars have demonstrated that 120 min of contact with nature per week leads to better health benefits (White et al., 2019). Similarly, our findings suggest that gardening twice or more per week enhances wellbeing outcomes.

The results showed that even weekly gardening significantly reduced stress levels compared to those who did not engage in gardening. This highlights the importance of accessible gardening spaces for urban populations with limited time. In the cultural context of Kazakhstan, where dacha and gardening are traditions, these benefits are likely enhanced by the connection to cultural identity and family traditions. However, 35.7% of the participants did not garden. The primary barriers among participants were a shortage of space (24.0%), time (20.0%), and lack of knowledge and skills (16.0%). Some reported motivational barriers included not wanting to take responsibility for the process (10.7%), lack of interest (6.7%), and high costs (6.7%). These findings suggest that the public is generally willing to participate if the main obstacles are addressed by supportive policies. Notably, half of the non-gardeners engaged in passive leisure activities, such as watching television (29.3%) and using social media (13.3%). Therefore, public health initiatives that turn barriers into accessible gardening opportunities could help displace these sedentary activities with a health-promoting alternative and offer valuable implications for urban wellbeing policies.

## 6 Conclusion

This study aimed to examine how gardening frequency and leisure orientation contribute to activity attachment, which leads to improvements in mental wellbeing and stress reduction. Through a comprehensive analysis, the results indicate that participants who engage in gardening report higher attachment to the activity, as well as higher mental wellbeing and lower perceived stress, compared to non-gardeners. This difference surpasses the variations in gardening frequency or leisure orientation among gardeners. In detail, in the present investigation, gardening frequency and leisure orientation directly affected enhancing activity attachment, mental wellbeing, and reducing perceived stress. Thus, when active attachment is high and positive among people, we find that the relationship between high frequency leisure orientation and mental wellbeing and reduced perceived stress can be positive and significant. The current study provides new knowledge on the various relationships between frequency, leisure, and active attachment, as well as mental wellbeing and stress among gardeners and non-gardeners in developing countries such as Kazakhstan. Initiatives can address the specific barriers identified in this study. For example, container gardening and community plot initiatives could dramatically expand participation for 24.0% of non-gardeners who indicated a lack of space. Messages and advertisements can emphasize that even weekly participation provides significant benefits for the 20.0% of respondents who reported a lack of time. Available skills development workshops, as cost-effective measures, can be applied to 16.0% of participants who lack the knowledge and skills. A new approach to developing leisure-related green gardening spaces would be beneficial for respondents who opt for high costs (6.7%) or are not ready to assume long-term responsibilities (10.7%). Given Shymkent’s favorable climate, seasonal and short-term community gardening programs could provide residents with practical opportunities to participate without requiring ongoing commitments. However, the scale of the differences between gardeners and non-gardeners vastly exceeds the within-gardener variations. Overall, the results suggest that increased participation and leisure-oriented gardening practices are associated with higher activity attachment, which in turn affects better mental wellbeing. The results suggest that public health resources should focus on expanding participation.

### 6.1 Theoretical contributions

The current research presents three interrelated theoretical contributions, all of which argue that activity attachment is a central concept in nature-based wellbeing studies. First, we extended attachment theory by introducing activity attachment as a novel construct in the gardening study. Previous work has focused on place attachment (Barrable, 2025; Scannell and Gifford, 2010); however, we validated that psychological bonds with an activity itself serve as a potential mediator for mental health benefits. It establishes activities as legitimate attachment objects for the elderly. Second, this study integrates leisure studies with environmental psychology. We found that intrinsic motivation from recreation research (Fox, 2017; Murtagh and Frost, 2023), which is measured as leisure orientation, significantly predicted activity attachment and wellbeing outcomes in developing countries. Third, the results found a dual-pathway model. SEM shows that frequent gardening for leisure purposes has a substantial direct effect on gaining immediate benefits and (b) an indirect effect through attachment, which gains additional benefits.

### 6.2 Integrated policy and planning recommendations

A key public health strategy is to reimagine gardening in public communication. This suggests that it can be transformed from a chore into a desired form of relaxation and rejuvenation. This reimagining aims to overcome the key barriers to participation related to time and motivation. Support the national dacha roadmap and infrastructure. It recognizes that it functions not simply as an agricultural program but as an essential public health measure. Its success in expanding access directly influences the improvement of the population’s mental health. Policymakers could develop a new green gardening sector, such as U-pick farms, as they are comparably low-cost and leisure-oriented activities. It has scientifically proven stress-reducing benefits.

### 6.3 Study limitations and future study directions

The present study has limitations that guide future research. A sample from a single city, such as Shymkent, can limit the ability to generalize the findings. Future studies should employ longitudinal designs and conduct studies in other cities across diverse settings. Our study did not test controlled confounding variables, which may have influenced the relationship between activity engagement and wellbeing. Future research should consider including relevant demographic, environmental, and psychological covariates to account for these effects. Moreover, some survey items were removed during the factor analysis because of low factor loadings. Although the instruments were adapted for local relevance, these modifications may limit comparability with prior studies and affect the measurement of constructs. A key methodological opportunity is a multi-group SEM analysis comparing our model between gardeners and non-gardeners. Testing for measurement and structural invariance will allow us to determine whether the strength of psychological relationships (from activity attachment to wellbeing) is similar across activities. This will allow us to determine whether these benefits are a universal function of a favorite leisure activity or whether they are specifically enhanced by gardening. Third, our findings show that almost half of the non-gardeners engaged in passive leisure activities. It stresses that future research should test gardeners and non-gardeners as two distinct categories. They can compare the wellbeing outcomes and underlying mechanisms of gardening with other activity categories, such as passive screen time, social leisure, and sports. Such a comparative approach will allow us to explore whether the benefits of gardening are related to a restorative connection with nature, an active and creative process, or a combination of both.

## Data Availability

The raw data supporting the conclusions of this article will be made available by the authors, without undue reservation.
